# Too much care? Increasing checkup frequencies and declining role of general practitioners in antenatal care in Norway (2010-2021)

**DOI:** 10.1080/02813432.2025.2575326

**Published:** 2025-10-22

**Authors:** Ragne Victoria Tonesdatter Kolaas Stauri, Kristine Pape, Bente Prytz Mjølstad, Kjartan Sarheim Anthun, Gunnhild Åberge Vie

**Affiliations:** ^a^ Department of Public Health and Nursing, Norwegian University of Science and Technology (NTNU), Trondheim, Norway; ^b^ Department of Health Research, SINTEF, Trondheim, Norway; ^c^ General Practice Research Unit, Department of Public Health and Nursing, Norwegian University of Science and Technology (NTNU), Trondheim, Norway

**Keywords:** Prenatal care, pregnancy, general practitioners, midwifery, Norway, demography, health planning guidelines, national health programs

## Abstract

**Introduction:**

The antenatal care program is a cornerstone of Norway’s national health programs, serving nearly 50 000 women annually. Although usage has risen since the 1980s, with antenatal checkups exceeding recommendations, research on utilization patterns and demography remains limited. Insight is essential for optimizing prenatal care services, containing costs, and avoiding potential overtreatment.

**Material and methods:**

This retrospective descriptive study used registry data from the Control and Payment of Health Reimbursement database, Statistics Norway, and the Norwegian Patient Register, covering 381 092 women giving birth in Norway (2010–2021). Associations were estimated using Poisson regression.

**Results:**

Between Jan 1, 2010, and Dec 31, 2020, the mean number of antenatal checkups increased from 11.1 to 13.0, exceeding guidelines by 5.0. Midwife checkups increased by 2.4, while general practitioner (GP) checkups decreased by 1.1, progressively making midwives the main providers. Compared to women with lower secondary education, highly educated women had 7% more checkups by midwives and 12% fewer with GPs. They also sought GPs less before, during, and after pregnancy (48%, 20%, and 28% less). Women with 16+ checkups (upper quartile) had lower education and 1.6 more GP consultations the year before pregnancy than those with fewer checkups.

**Conclusion:**

From 2010 to 2021, GPs saw a decrease in their role as main providers of antenatal checkups, with midwives now conducting most examinations. The average number of checkups rose by 1.9, not attributable to guideline changes, raising concerns about overutilization among a generally healthy group of women and implications for health system sustainability.

## KEY MESSAGE

From 2010–2021, general practitioners’ role in antenatal care declined as midwives became primary providers. Still, antenatal checkups increased by 1.9, not attributable to guideline changes, raising concerns about potential overtreatment in a generally healthy group of the population.

## Graphical Abstract

**Figure:**
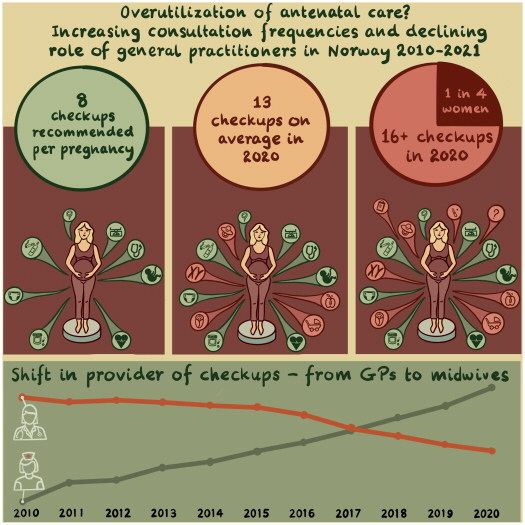

## Introductions

Preventive healthcare consultations during pregnancy - antenatal checkups - became common in Norway in the 1930s [1]. Despite this, official guidelines were not established until 1984, recommending 10 checkups for first-time pregnancies and six for subsequent ones [1]. Today, Norway offers an extensive antenatal care program to all pregnant women, including postpartum checkups, free of charge, averaging 57 000 participants annually between 2010 and 2021 [2].

Norway′s healthcare system is divided into primary and specialized care [3]. Primary antenatal care is provided by both GPs and municipal midwives, both qualified to provide routine antenatal checkups, with no strict division of responsibilities. However, midwives cannot prescribe (except contraceptives and vaccines) or issue sick leave. Each checkup includes clinical assessments (e.g. blood pressure, urine tests, fetal growth) and addresses the woman’s overall well-being [4].

Some midwives have lab access, others rely on GPs for blood tests and follow-up. Women choose how to divide checkups between GPs and midwives, though many municipalities recommend alternating between providers, e.g. to ensure GP checkups at the recommended blood sampling intervals. Antenatal checkups are not limited to only scheduled screenings and examinations. Antenatal checkups also address individual concerns, with extra checkups/tests offered if needed. Both GPs and midwives can refer to specialist care, which, unless urgent (e.g. delivery or reduced fetal movements), is otherwise inaccessible without referral.

**Figure 1.**
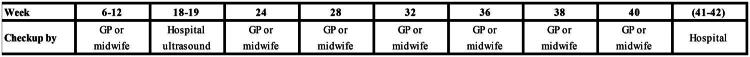
National guideline recommendations for antenatal checkups in Norway 2010–2021.

Some changes have been implemented to the program since 1984, such as the incorporation of week 16–18 routine ultrasound in 1986 and early ultrasound in 2022 [5,6]. Throughout the study period (2010–2021), the national guidelines (Figure 1) recommended eight antenatal checkups for normal full-term pregnancies; including seven checkups with a GP or midwife and one ultrasound examination [4]. In recent years, large political efforts have been made to enhance the municipal midwifery services in Norway [7]. In 2016, as part of this initiative, midwives were also authorized to prescribe long-acting reversible contraception.

The antenatal care program is comprehensive, requiring significant resources and financial costs for municipalities and the healthcare system to provide services to a generally healthy pregnant population. Still, research on this program is limited. Despite the recommendation of eight checkups, the few studies conducted between 1988 and 2000 showed an average number of checkups ranging from 12.0 to 12.7, not including postpartum checkups [8–10]. These studies were based on smaller cohorts of women. A report using registry data from 2015 to 2017 found Norwegian women averaged about 12 checkups per pregnancy [11]. Our study is the first to use large-scale registry data to examine antenatal care utilization in Norway and at the same time explore how various sociodemographic groups utilize the service and identify frequent users.

The hypothesis suggests that antenatal care utilization exceeds guidelines, and that improved midwifery access influences usage patterns. It also proposes that different sociodemographic factors among women result in varying levels of service utilization. We aimed to study women who gave birth in Norway over a decade using large registry data. The objective was to investigate if the frequency of checkups aligns with guidelines, how different sociodemographic groups utilize antenatal care, and to describe trends over time.

## Material and methods

### Study design, setting, and data sources

This study was a registry data-based retrospective descriptive study that included all women in Norway who gave birth between January 1, 2010, and December 31, 2020. We extracted primary care data on GP and midwife checkups from the KUHR database (Control and Payment of Health Reimbursement), sociodemographic variables from Statistics Norway (SSB), and specialist healthcare contacts and hospital admissions from the Norwegian Patient Registry (NPR). A unique personal identifier enabled the linkage of data from various sources.

### Participants and study sample

We identified 570 142 births in 388 279 women from hospital records based on DRG codes. The dataset lacked precise birth dates for the children. Consequently, the birth date was assigned as either the mother’s hospital admission date or, when available, the date linked to pregnancy and childbirth-related procedural codes (Supporting Information Table S1). Supporting Information Figure S1 shows how we defined the study sample: multiple birth admissions within 14 days were considered the same event, selecting the first admission. We excluded 2 381 births due to the inability to confirm the birth date, as the interval between two registered births ranged from 15 to 280 days. To avoid analyzing healthcare contacts related to earlier pregnancies, births were excluded if the previous delivery was less than two years (730 days) prior. For the analyses of associations, we only included the first birth for multiparous women. For analyses of time trends, we included the first birth per year per woman, to avoid bias from including fewer multiparous women towards the end of the period. Women who immigrated and emigrated with less than one month of observation time, as well as terminated pregnancies and miscarriages, were excluded. Our study ultimately included 380 916 births/women in the analysis of associations and 511 450 births among 381 092 women in the time trend analyses.

### Time observed

We defined three observation periods for each woman: one year before, during, and one year after pregnancy. Data were structured as repeated gestational monthly observations, with each month standardized to 30 days, to facilitate analysis. Each woman was observed for 990 days: 360 days pre-pregnancy, 270 days during pregnancy, and 360 days post-pregnancy. Only the months in which the woman was registered as a citizen in Norway were included in the analyses. As a result, 1 770 women did not contribute with observation time before or during pregnancy.

### Missing data

From 2010 to 2021, SSB recorded 637 171 births, whereas our dataset listed 570 142, indicating 67 029 (10.5%) missing births. This discrepancy likely arises from women being hospitalized for non-childbirth reasons and staff failing to include a separate DRG code for delivery. Transport and home births (0.91-0.95% annually) might also explain a portion of unrecorded births [12]. Regarding covariates, data were missing for 16 539 women for educational level, 1 for immigration status, and 9 038 for municipality population.

### Outcomes and consultation categories

In our study, we defined two types of categories of health care encounters: 1) antenatal checkups and 2) consultations with GPs for any reason. The codes used to identify these are specified in Supporting Information Table S1.

Antenatal checkups are defined as consultations related to preventative health care/screening in pregnancy, including both routine checkups recommended for all pregnant women and additional checkups on indication. This definition follows the approach used in previous Norwegian studies. In order to compare overall use of GP service in the year before, during and following pregnancy, we also included a broader category: GP consultations for any reason. This includes all GP consultations, regardless of whether they were related to pregnancy. Antenatal checkups, as defined above, are a subset of this broader category.

To define antenatal checkups by GPs and midwives, we used data from the reimbursement system (KUHR). Antenatal checkups were identified as: (1) all consultations with reimbursement code 217a/b (antenatal/postpartum checkups by GPs) or 1a/b (checkups by municipal midwives), regardless of accompanying diagnostic codes. Because the reimbursement system only permits the use of code 217a/b six times per pregnancy, we also included: (2) all consultations with ICPC-2 diagnostic code W781 (‘antenatal checkup’), regardless of accompanying diagnostic codes, and: (3) all consultations with ICPC-2 diagnostic code W78 (‘confirmed pregnancy’), but only when W78 appeared as the sole diagnostic code. The restriction for W78 was applied to minimize misclassification, as W78 is often used for administrative purposes (e.g. to exempt co-payment in pregnancy).

The distinction between antenatal checkups and clinically indicated consultations during pregnancy is not always well defined. To isolate antenatal checkups as a separate category representing screening and preventive care, we did not include consultations coded for treatment or follow-up of pregnancy complications (e.g. gestational diabetes, preeclampsia), unless they also fulfilled the three above-mentioned criteria for antenatal checkups. These consultations, however, remain included in the category of ‘GP consultations for any reason’.

Doctors working at local out-of-hours medical centers often also serve as GPs. However, checkups at these centers were categorized as checkups by ‘others’ and not as checkups by GPs. The codes used to define checkups in the secondary health service are also available in Supporting Information Table S1.

We analyzed four types of healthcare encounters during observation periods before, during, and after pregnancy:

1. antenatal/postpartum checkups performed by GPs
2. antenatal/postpartum checkups performed by municipal midwives
3. antenatal checkups performed by others; specialists, outpatient clinics, and local out-of-hours medical centers
4. consultations with GPs for any reason, including antenatal checkups

Antenatal and postpartum checkups were identified using the same codes but were separated by their timing relative to birth.

Due to the lack of a parity variable in the KUHR database, we could not differentiate between first-time and multiparous mothers. To evaluate potential differences in checkups related to parity, we performed a sensitivity analysis comparing women who gave birth in 2019 with those who had no, one, or two previous births between 2010 and 2018. For women who delivered in 2019 without prior births recorded between 2010 and 2018, we presumed primiparity, as any earlier births would have occurred at least nine years earlier.

### Covariates

The study examined maternal age, educational attainment, immigration status, and municipality population, with corresponding categories, as shown in Tables 1 and 2. Here, municipality refers to the woman’s registered residence, not the location of the healthcare provider. However, these locations often overlap, as many women receive care close to their place of residence.

**Table 1.** Incidence rate ratio (IRR), 95% CI: the impact of sociodemographic factors on GP consultations (for any reason) before, during, and after pregnancy.

	(*n* = number of women)	The year before pregnancy		During pregnancy		The year after pregnancy	
Highest level of education	**Lower secondary education (13–15 yo) or lower** (*n = 70 413*)	1.00		1.00		1.00	
	**Upper secondary education (16–19 yo)** (*n = 92 724*)	0.83	(0.83,0.84)	0.97	(0.97,0.98)	0.88	(0.88,0.89)
	**Bachelor’s degree** (*n = 137 787*)	0.65	(0.65,0.65)	0.89	(0.88,0.89)	0.79	(0.78,0.79)
	**Master’s degree or higher** (*n = 62 638*)	0.52	(0.52,0.52)	0.80	(0.79,0.80)	0.72	(0.72,0.73)
Maternal age	***≤* 19** (*n = 4 684*)	0.82	(0.80,0.83)	0.86	(0.85,0.87)	0.98	(0.97,1.00)
	**20–24 ** *(n = 47 215)*	0.97	(0.97,0.98)	0.98	(0.98,0.99)	1.02	(1.01,1.02)
	**25–29 ** *(n = 120 202)*	1.00		1.00		1.00	
	**30–34 ** *(n = 126 206)*	1.04	(1.03,1.04)	0.99	(0.99,0.99)	0.98	(0.97,0.98)
	**35–39 ** *(n = 63 583)*	1.10	(1.10,1.11)	1.00	(1.00,1.01)	1.00	(0.99,1.00)
	***40–44*** *(n = 15 954)*	1.20	(1.19,1.21)	1.01	(1.01,1.02)	1.05	(1.04,1.06)
	***45 ≤*** *(n = 1 302)*	1.29	(1.25,1.33)	1.03	(1.01,1.05)	1.09	(1.06,1.12)
Immigration status of mother	**Norwegian born with two Norwegian parents** (*n = 256 111*)	1.00		1.00		1.00	
	**Norwegian born with one Norwegian parent** (*n = 15 893*)	1.01	(1.00,1.02)	1.02	(1.01,1.02)	1.01	(1.01,1.02)
	**Norwegian born with immigrant parents** (*n = 4 991)*	1.21	(1.19,1.22)	1.20	(1.19,1.21)	1.12	(1.11,1.14)
	**Immigrant** (*n = 102 150*)	0.88	(0.88,0.89)	1.08	(1.08,1.08)	0.93	(0.92,0.93)
Municipality population	**Municipality ≤ 4 999 inhabitants** (*n = 31 092*)	1.00		1.00		1.00	
	** Municipality 5 000–19 999 inhabitants** (*n = 91 785*)	1.06	(1.05,1.07)	1.18	(1.17,1.18)	1.06	(1.05,1.06)
	**Municipality ≥ 20 000 inhabitants** (*n = 248 918*)	1.06	(1.05,1.07)	1.32	(1.32,1.33)	1.09	(1.08,1.10)

Adjusted for educational attainment, maternal age, immigration status, and municipality population.

**Table 2.** Incidence rate ratio (IRR), 95% CI: the impact of sociodemographic factors on the utilization of antenatal checkups (excluding postpartum checkups) by midwives and/or GPs.

	(*n* = number of women)	Midwife + GP		Midwife		GP	
Highest level of education	**Lower secondary education (13–15 yo) or lower** (*n = 70 413*)	1.00		1.00		1.00	
	**Upper secondary education (16–19 yo)** (*n = 92 724*)	1.00	(1.00,1.01)	1.03	(1.02,1.03)	0.99	(0.98,0.99)
	**Bachelor’s degree** (*n = 137 787*)	0.99	(0.99,0.99)	1.08	(1.07,1.08)	0.92	(0.92,0.93)
	**Master’s degree or higher** (*n = 62 638*)	0.96	(0.96,0.96)	1.07	(1.06,1.08)	0.88	(0.87,0.88)
Maternal age	***≤* 19** (*n = 4 684*)	1.01	(1.00,1.02)	1.11	(1.10,1.13)	0.93	(0.91,0.94)
	**20–24 ** *(n = 47 215)*	1.02	(1.02,1.03)	1.05	(1.05,1.06)	0.99	(0.99,1.00)
	**25–29 ** *(n = 120 202)*	1.00		1.00		1.00	
	**30–34 ***(n = 126 206)*	0.96	(0.96,0.96)	0.91	(0.91,0.91)	1.00	(1.00,1.01)
	**35–39 ***(n = 63 583)*	0.93	(0.93,0.93)	0.81	(0.81,0.82)	1.03	(1.03,1.04)
	**40–44 ***(n = 15 954)*	0.91	(0.90,0.91)	0.77	(0.76,0.78)	1.03	(1.03,1.04)
	**45 ≤** *(n = 1 302)*	0.85	(0.83,0.87)	0.69	(0.67,0.71)	0.99	(0.96,1.01)
Immigration status of mother	**Norwegian born with two Norwegian parents** (*n = 256 111*)	1.00		1.00		1.00	
	**Norwegian born with one Norwegian parent** (*n = 15 893*)	1.00	(1.00,1.01)	1.00	(0.99,1.01)	1.01	(1.00,1.01)
	**Norwegian born with immigrant parents** (*n = 4 991)*	1.02	(1.02,1.03)	1.01	(1.00,1.03)	1.04	(1.02,1.05)
	**Immigrant** (*n = 102 150*)	1.04	(1.03,1.04)	0.97	(0.97,0.97)	1.09	(1.09,1.09)
Municipality population	**Municipality ≤ 4 999 inhabitants** (*n = 31 092*)	1.00		1.00		1.00	
	**Municipality 5 000–19 999 inhabitants** (*n = 91 785*)	1.22	(1.21,1.22)	1.11	(1.10,1.12)	1.35	(1.34,1.36)
	**Municipality ≥ 20 000 inhabitants** (*n = 248 918*)	1.27	(1.26,1.27)	0.98	(0.98,0.99)	1.64	(1.63,1.65)

Adjusted for educational attainment, maternal age, immigration status, and municipality population.

### Statistical analysis

First, we calculated the average number of antenatal checkups among pregnant women per year for GPs, municipal midwives, obstetric specialists, local out-of-hours medical centers, and hospital outpatient departments. Secondly, we calculated the monthly proportion of women attending antenatal checkups with different providers, comparing 2010 to 2020. Further, we used Poisson regression models to estimate the incidence rate ratio (IRR, 95% confidence interval) of a) GP consultations for any reason in the three observation periods and b) antenatal checkups with a GP, a midwife, or either of these. These analyses were mutually adjusted for age group, educational level, immigration status, and municipal population. To compare women with 0–15 vs 16+ antenatal checkups (upper quartile), we calculated their demographic characteristics, the frequency of GP consultations the year before pregnancy, and their distribution of ICPC-2 diagnostic codes. In sensitivity analysis, we examined time trends excluding women with high risk-pregnancies, preeclampsia and gestational diabetes, identified by ICPC-2 diagnostic codes W76, W81, W84, and W85. We also examined the average number of primary care antenatal checkups, among women who saw GPs only, midwives only or a combination of both, respectively. Statistical analyses were conducted using Stata version 18.

## Results

**Figure 2.**
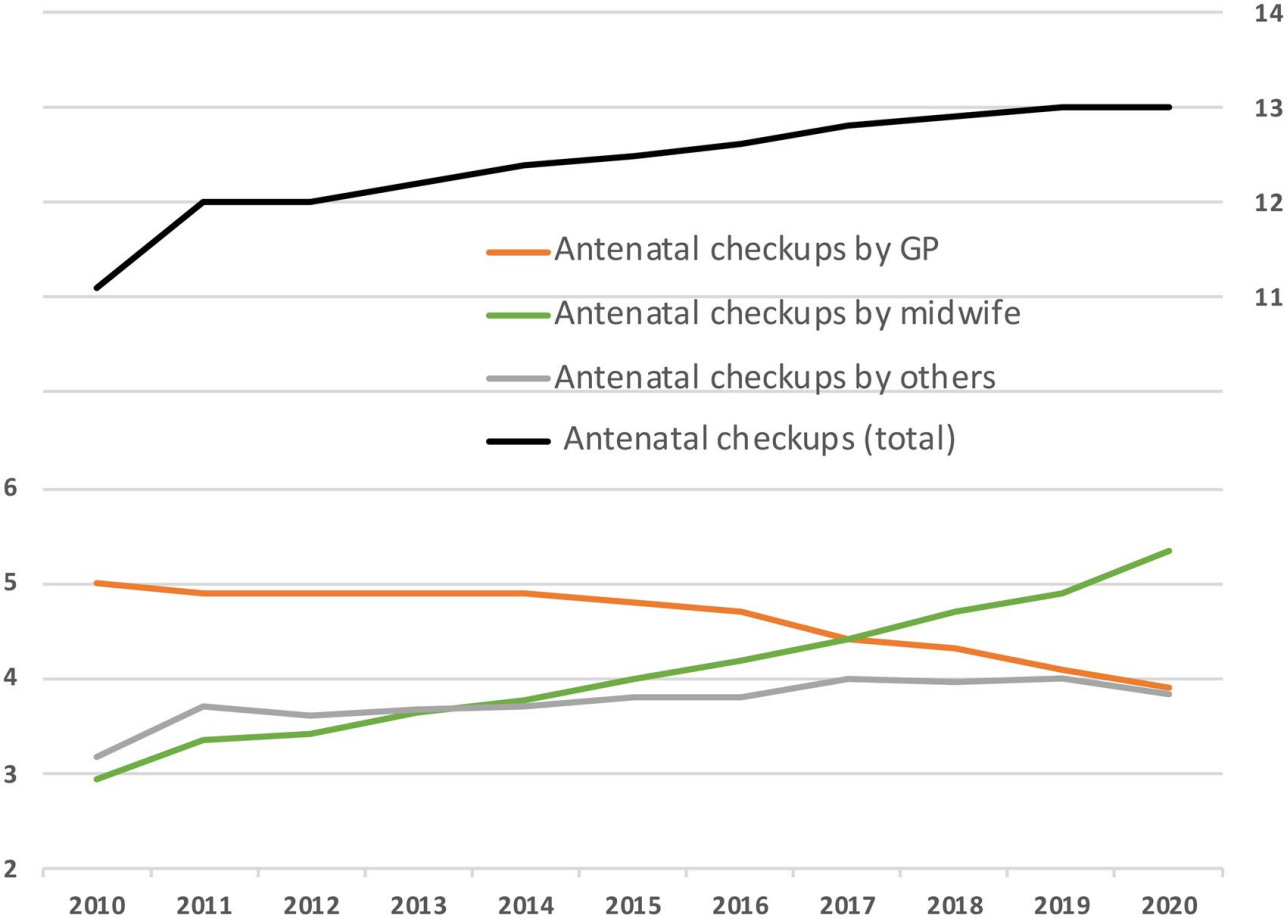
Mean number of antenatal checkups per woman per year by health care provider, from 2010 to 2021.

Our total dataset included 7 540 086 antenatal/postpartum checkups and 7 050 451 GP consultations (including antenatal/postpartum checkups by GPs) related to 511 450 births among 381 092 women from Jan 1, 2010, to Dec 31, 2020. During this period, the average number of antenatal checkups per pregnancy, including both primary and specialized care, rose from 11.1 to 13.0, surpassing guidelines by 5.0 appointments. Postpartum checkups came in addition to these. The median number of checkups rose from 11.0 to 12.0. While the number of antenatal checkups by GPs decreased over time, the number of GP consultations during pregnancy for any reason (including antenatal checkups) increased from 7.5 in 2010 to 8.2 in 2020. Figure 2 shows the mean number of antenatal checkups per woman per year (details in Supporting Information Table S2). Sensitivity analysis of the presumed primiparous women who delivered in 2019 with no prior births from 2010 to 2018 showed an average of 13.8 checkups, compared to 13.6 and 13.2 for women with one and two prior births in the same period. During the study period (2010–2021), the primary providers of antenatal checkups shifted from GPs to midwives. On average, midwives increased by 2.4 checkups (2.9–5.3), and GPs decreased by 1.1 (5.0-3.9). Other services contributed an increase of 0.7 checkups on average. This included hospital checkups (*n* = 1 691 499), specialist checkups (*n* = 211 186), and checkups at local out-of-hours medical centers (*n* = 6 687). Sensitivity analysis excluding women with high-risk pregnancies, preeclampsia, or gestational diabetes, showed only slightly lower estimates with similar time trends (details available in Supporting Information Table S3).

**Figure 3.**
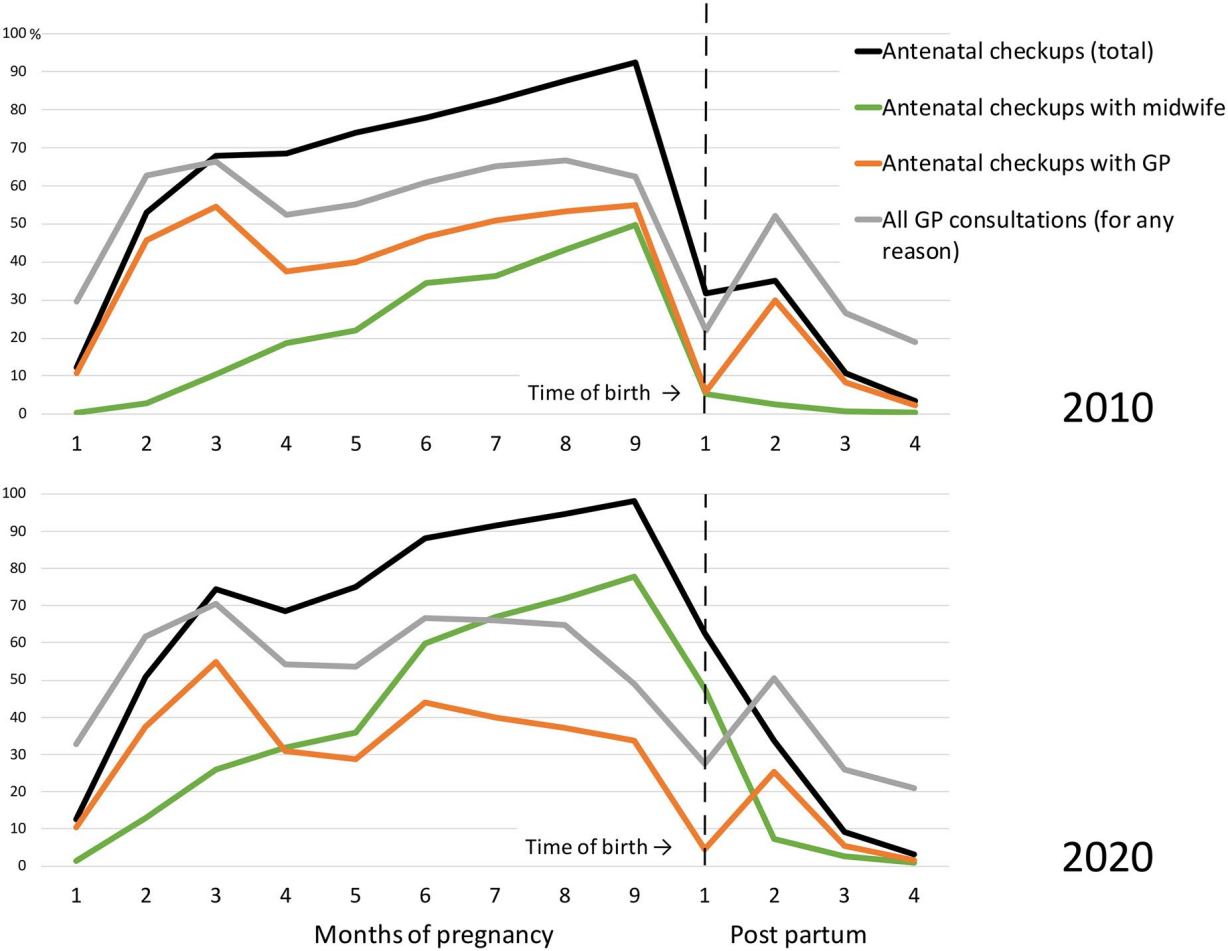
The proportion of women with GP consultations (for any reason) and antenatal checkups per gestational month. The total percentage of antenatal checkups per month (black line) includes all checkups performed by GPs, municipal midwives, obstetric specialists, local out-of-hours medical centers, and hospital outpatient departments.

When assessing health care contacts among the study participants throughout the three observation periods, the proportion with GP consultations per month was relatively stable the year prior to pregnancy and from four months postpartum. Therefore, Figure 3 only highlights the proportion of women with a GP consultation or antenatal checkup during pregnancy and four months postpartum, comparing 2010 to 2020. In 2010, an average of 19.3% of women visited their GP (regardless of reason) each month in the year preceding pregnancy. By 2020, this proportion had increased to 21.3%. Four months postpartum, GP consultations returned to pre-pregnancy levels. One year after birth, the frequency of GP consultations remained unchanged compared to one year before pregnancy, with approximately one in five women having a GP consultation per month. From 2010–2021, participants averaged 2.87 GP consultations in the 12-month period before pregnancy, 2.88 consultations during pregnancy (excluding antenatal checkups), and 2.67 consultations in the year after (median = 2 for all three periods).

This study covers the first year of the COVID-19 pandemic. However, there were only marginal differences in the antenatal checkup rates between 2019 and 2020. From 2010 to 2020, there was a consistent increase in the mean number of antenatal checkups per pregnancy conducted by midwives and a corresponding decrease in checkups performed by GPs. In 2010, GPs conducted the largest proportion of checkups each month throughout pregnancy, with 55% of women having a checkup by their GP in the ninth month of pregnancy. By 2020, this proportion had sunk to 34%, and midwives had assumed the role of the professional group performing the clear majority of checkups from the fourth month of pregnancy onwards. In 2020, 78% of women had at least one antenatal checkup with a municipal midwife in the ninth month of pregnancy, compared to 50% in 2010.

A clear difference was observed in the utilization of postpartum checkups between 2010 and 2020. The proportion of midwife checkups increased substantially, whilst GP checkups declined. In the first and second months after birth in 2010, 5.3% and 2.4%, respectively, had checkups with a municipal midwife. By 2020, these figures had increased to 47.7% and 7.4%. The corresponding figures for GPs were 5.9% and 30.0% in 2010, and 4.5% and 25.2% in 2020 (Figure 3).

Table 1 illustrates the impact of sociodemographic factors on GP consultations (for any reason). Educational level was strongly associated with GP use. Women with a master’s degree consulted the GP considerably less often than those with lower secondary education – about half as often the year before pregnancy, somewhat less often during pregnancy, and roughly one third less in the year after birth. Maternal age was positively associated with GP consultations before pregnancy, with the oldest age groups (40 <) consulting considerably more often than those aged 25–29. This pattern persisted, though less pronounced, during and after pregnancy. Women of immigrant backgrounds had more GP consultations during pregnancy compared to Norwegian-born women. However, first-generation immigrants consulted less frequently both before and after pregnancy. Finally, women residing in municipalities with more than 20,000 inhabitants had around one third more GP visits during pregnancy than those in the smallest municipalities (Table 1).

Table 2 demonstrates the influence of sociodemographic factors on the utilization of antenatal checkups by municipal midwives and GPs. Overall, education had very little impact on the total number of antenatal checkups. However, women with higher education more often chose midwives over GPs. Maternal age was negatively associated with the number of checkups: women above 35 had fewer antenatal checkups than those aged 25–29, particularly due to a substantial reduction in midwife checkups. Antenatal checkups were also more frequent in larger municipalities, with women in the most urban areas attending far more often than those in the smallest municipalities, especially in terms of GP checkups. Immigration status, by contrast, had little effect on the use of either midwife or GP antenatal checkups (Table 2).

One fourth of the study population underwent 16 or more antenatal checkups. This group of frequent users had 1.6 more GP consultations on average the year before pregnancy compared to all others (4.3 vs 2.7). They also had lower educational attainment (49.2% vs 41.8% had only finished upper secondary education) and a slightly higher proportion of immigrants (23.8% vs 22.5%). In the year before pregnancy, frequent users had a higher prevalence of diagnoses from all chapters in the ICPC-2 compared with all others, with most notable disparities in the diagnostic groups ‘General and unspecified’ (31.7% vs 25.9%), ‘Digestive’ (21.8% vs 14.3%), ‘Psychological’ (31.9% vs 25.1%), ‘Musculoskeletal’ (29.2% vs 21.2%), and ‘Other’ (64.6% vs 51.2%). Further details are provided in Supporting Information Table S4.

**Figure 4.**
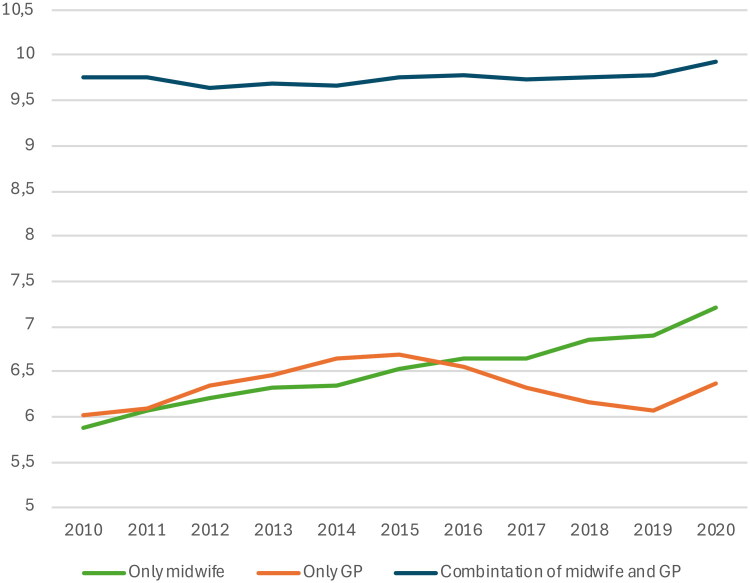
Mean number of antenatal checkups with a GP or midwife, among women who only saw a midwife, only a GP, or a combination of both, as their primary care provider (irrespective of use of specialized health services), from 2010 to 2021.

Figure 4 shows the mean number of antenatal checkups for women who received checkups exclusively from midwives, GPs, or both providers combined. Women with checkups exclusively from midwives showed a steady increase from 5.9 checkups in 2010 to 7.2 in 2020, while those seeing only GPs (ranging from 6.0 to 6.7) or both providers (ranging from 9.6 to 9.9) showed no such trend. The proportion receiving antenatal care solely from midwives increased from 3.2% in 2010 to 8.6% in 2020. Further details are provided in Supporting Information Table S5.

## Discussion

The findings of this study reveal a steady increase in the total number of antenatal checkups in Norway from 2010 to 2021, with a rise from 11.1 to 13.0 checkups on average per pregnancy, exceeding national guidelines by 5.0 appointments. Midwives progressively assumed a primary role in antenatal care provision, while the involvement of GPs declined. At the same time, the overall utilization of GP consultations (for any reason) increased slightly. Sociodemographic variations in care utilization were modest but notable. An average of 13.0 antenatal checkups represent the highest mean number of checkups identified in studies since the implementation of the antenatal program in Norway [8–11]. The underlying cause is not readily apparent. Based on our findings, guideline changes, such as altered glucose tolerance testing recommendations in 2017, did not noticeably impact usage the following year. On the contrary, utilization increased steadily throughout the study period. The rise may to some extent be linked to increasing maternal age, as the average age of first-time mothers increased from 28.1 to 29.9 from 2010 to 2020 [2]. Still, age-related morbidities are unlikely to explain this trend alone, as fertile women still represent a relatively healthy cohort of women.

Increasing BMI or worsening maternal health during the study period may have contributed to a higher prevalence of pregnancy-related complications, and therefore an increase in the number of checkups. However, our findings do not strongly support this hypothesis. Although there was a modest increase in the number of antenatal checkups in specialist care, suggesting a slight rise in higher-risk pregnancies, the overall pattern does not indicate a general increase in maternal morbidity. If worsening maternal health were the primary driver of increased service use, we would expect a corresponding increase in GP antenatal checkups as well, as midwives in Norway cannot prescribe medications, issue sick leave, or manage certain medical concerns that require doctor follow-up. Instead, our results show stable consultation rates with GPs for any reason until 2019, a decline in GP antenatal checkups, and an increase in midwife checkups, suggesting other factors, such as improved access to midwifery services, changing models of care, and increasing demand for reassurance, may better explain the trend. Besides, in our sensitivity analysis of time trends, where we excluded women with high-risk pregnancies, preeclampsia, and gestational diabetes – conditions often linked to increasing maternal age and BMI – we still observed the same trend of increased use.

The official recommendation during the study period was eight checkups for a full-term pregnancy, with the option of additional follow-up if needed [4]. Therefore, no upper limit exists for the total number of checkups. An average of 5.0 additional antenatal checkups per woman may still suggest overutilization.

Our definition of antenatal checkups as a category representing screening and preventive care depends on accurate coding and a shared understanding with GPs of what constitutes a checkup. If some GPs failed to add an antenatal checkups code, this may have led to a slight underestimation of GP-provided antenatal checkups. Nevertheless, all consultations are captured in the broader category of ‘GP consultations for any reason.’ While total GP consultations increased slightly (0.7), GP-provided antenatal checkups declined, whereas midwife-provided checkups increased substantially – more than three times the increase in total GP use. This indicates that pregnant women are using services more overall, but with a clear shift in provider for antenatal care from GPs to midwives.

During this period, midwifery services expanded across Norwegian municipalities, with a 5-15% increase in midwife person-years from 2015 to 2020 [7]. In addition, since private midwife checkups, a popular service in several Norwegian regions for extra checkups and private ultrasounds, were not recorded in our data, the actual number of antenatal checkups was likely higher. The shift toward midwives handling more antenatal care tasks may improve access but raises concerns about continuity and coordination. Kinge and Grytten (2021) found that higher GP density correlates with better perinatal outcomes, highlighting the importance of GP expertise and reinforcing their vital role in maternal and infant health [13].

It is worth noting that one in four women underwent 16 or more checkups per pregnancy in 2020. These women also had approximately 1.6 more GP consultations in the year before pregnancy. One possible explanation is that these women had a higher prevalence of pre-existing conditions, necessitating more intensive monitoring during pregnancy. However, the differences between the groups were evident across nearly all diagnostic categories. It so appears that frequent GP consultations in the year before pregnancy, regardless of the diagnostic category, were strongly associated with an increased likelihood of excessive antenatal checkups.

The observed increase in antenatal checkups may reflect a broader trend of rising healthcare utilization among women and younger individuals, rather than being a unique phenomenon. Pregnancy is a period where psychological well-being, health behaviors, expectations of care, and the ability to manage uncertainty are relevant; factors that may be amplified in an era of digitalization and extensive social media exposure, where pregnant women can experience an overwhelming influx of information, which may prompt more worries, causing frequent reassurance-seeking through medical consultations [14,15]. The increase in antenatal checkups among women attending only midwife checkups, versus relatively stable numbers among those seeing only GPs or both providers, suggests that changes in antenatal care utilization may be shaped more by evolving preferences and care models, reflecting a time trend where expanded access itself generates demand, rather than by increasing medical need. While improved access to midwifery services likely contributed to a growing proportion of women receiving midwife-only care, this does not explain the rise in the number of checkups within this group. The finding may reflect a greater demand for reassurance and supportive care, or a tendency toward closer follow-up in midwife-led models. Longer consultation times with midwives may also make them attractive to women seeking more time for discussion and continuity.

When assessing postpartum checkups, it is important to note that the GP reimbursement system accepts the use of code 217a/b a maximum of six times per pregnancy, postpartum checkups included. Additional checkups are reimbursed as regular consultations, as suggested by the high GP consultation rates within the first two months postpartum. Municipal midwives often make home visits within three days of discharge from hospital. This is particularly relevant after early discharge and may contribute to an increased number of postpartum checkups in the first month after birth. Postnatal baby checkups by public health nurses should not be included in the dataset. However, misregistration of codes or timing of checkups is possible and might lead to overestimation of postpartum checkups. Still, postpartum checkups appear widely utilized and have increased substantially from 2010 to 2021, with midwives accounting for most of this increase.

The choice of healthcare provider varied across sociodemographic groups, with maternal age and educational level being key influencing factors. The women attended fewer antenatal checkups by GPs or midwives as they aged, and older mothers were more likely to seek GPs the year before pregnancy, possibly due to fertility concerns. Older mothers may have more pregnancy knowledge from prior births, better access to private healthcare due to financial stability, or a greater need for specialist follow-up, which may result in more checkups by specialist care rather than primary care services. Educational level also considerably influenced GP consultations for any reason before, during, and after pregnancy. Still, it is reassuring that the use of antenatal checkups was comparable across sociodemographic groups. Despite previous research highlighting barriers to antenatal care for recently immigrated women or undocumented immigrants [16,17], we found no indication that women with immigrant backgrounds in general attended fewer checkups than those with Norwegian-born parents.

Somewhat surprisingly, women with higher education levels preferred midwives to GPs for antenatal checkups. Higher-educated women may be more skilled at navigating the healthcare system, leading them to seek specialized care [18]. Midwives, offering specialized expertise in pregnancy and childbirth, may be considered more suitable for antenatal care. This interpretation is supported by a recent study showing that women with better self-reported health, often linked to higher socioeconomic status, were more likely to receive antenatal care solely from a midwife, while those with poorer health more often saw a GP [19]. Together, these findings suggest that midwife-led care is more common among healthier, higher-educated women, whereas GPs may be more involved in care for women with complex needs.

Women with higher education consulted their GP significantly less frequently (for any reason) the year before, during, and the year after pregnancy, consistent with the well-established socioeconomic gradient of health [20]. This may be because they have better health awareness, reducing the need for medical follow-up. Additionally, greater financial stability may allow access to private healthcare, thereby reducing their need for public GP services. However, when examining the total use of antenatal checkups, differences by education level were minimal.

Unlike previous studies, which mainly relied on surveys and self-reported data, this study utilized large registry data to calculate the number of checkups, enhancing the validity of the results. Although the registries are of high quality [21], we may still have misclassified ordinary GP consultations as checkups or vice versa. Another limitation of our study was the absence of a parity variable in the KUHR database; consequently, we had to construct a proxy measure utilizing women who had at least nine years since any previous birth, whom we therefore presumed to be primiparous. However, the difference in the number of checkups between zero, one, and two previous children was minimal. This finding aligns with previous studies reporting checkup averages of 12.4 vs 11.7 (2002) and 11.0 vs 10.6 (1994) for primiparous and multiparous women, respectively [8,10]. Another limitation of this study is the lack of qualitative data that could provide deeper insights into the reasons for the increased number of antenatal checkups. However, the proportion of missing data was low, which likely minimally affected the results. An additional limitation is the lack of data on gestational age at birth. Approximately 94% of births in Norway are full term [22], while the remaining 6% are preterm and likely associated with fewer antenatal checkups. Although birth admission dates were available, we had no information on due dates or gestational week at delivery. Estimating pregnancy length based on the timing of routine ultrasound scans was not feasible, as the scan is typically performed in weeks 16–18, but varies widely between individuals and regions and may occur earlier or later for clinical reasons. Thus, we were unable to adjust for pregnancy duration in our analyses. Including these preterm births may therefore have led to a slight underestimation of the average number of checkups.

The findings of this study highlight the need for healthcare policymakers to reassess and potentially revise antenatal care protocols to reduce unnecessary checkups. This aligns with the conclusions of the Norwegian Health Personnel Commission (2021), which highlighted the need to address workforce shortages and increasing competition for midwives, as midwifery services have increased within municipalities [23]. Given that guidelines recommend eight checkups for a normal full-term pregnancy, an average of 13.0 checkups may suggest overutilization. Understanding the nature of these checkups is essential for optimizing resource allocation.

Increased access to antenatal care may benefit women, even if not strictly medically indicated or economically justified. Reduced stress and greater reassurance could aid both mother and fetus. However, improved health outcomes due to an increasing number of checkups are uncertain, and public funding for healthcare services not deemed medically essential is a debatable subject, warranting further research. A Cochrane review found no heightened risk for mothers or babies with fewer checkups (around eight) in high-income countries [24]. With approximately 50 000 births annually in Norway, even one excess checkup per pregnancy results in an additional 50 000 checkups, incurring significant costs. Although our data suggest a rise in the number of antenatal checkups beyond recommended levels, we cannot determine the clinical necessity of each encounter. The designation of some checkups as ‘unnecessary’ is based on guideline discrepancies, not on individual assessments. Addressing the issue of potential overuse is essential for managing public health expenditures and ensuring that this increase reflects a genuine need for care rather than overutilization.

Ensuring future sustainability in healthcare services requires careful resource distribution. Consequently, minimizing non-essential antenatal checkups and overutilization is not only a matter of cost-effectiveness but also a necessary measure to preserve workforce capacity and ensure a robust healthcare system. Optimizing collaboration between GPs and midwives could enhance efficiency, as they are complementary rather than competing healthcare providers. A shared digital maternity record, which Norway lacks as of 2025, documenting previous and forthcoming examinations, would be essential in facilitating such cooperation. Once implemented, it would also greatly enhance research opportunities by allowing for more detailed analyses of individual care pathways, such as why some women choose midwife-only or GP-only care, or why the number of checkups was by far largest among women who opted for both GPs and midwives to conduct their checkups, indicating that a lack of coordination might be a reason for the higher-than-recommended number of checkups, although it does not explain the time trends.

## Conclusion

The findings of this study suggest an overuse of antenatal checkups, with the average number of checkups exceeding the guidelines by 5.0 appointments in 2020, and one in four women having 16 checkups or more during pregnancy. This rise coincides with a shift in primary care providers from GPs to midwives, alongside noteworthy sociodemographic disparities in service utilization. This trend raises concerns about overtreatment in a generally healthy population, highlighting the need for healthcare providers and policymakers to consider targeted interventions to optimize antenatal care and ensure efficient resource use. Future research should investigate the underlying factors driving this rise and explore its potential impact on other areas of healthcare utilization in relation to pregnancy, such as pre-existing conditions, the utilization of referrals and sick leave, including whether women who are granted sick leave have a higher frequency of antenatal checkups. Further, studies should examine whether increased use leads to better health outcomes for mothers and babies.

## Supplementary Material

Graphical Abstract Figure.jpg

Supporting Information Table S5.docx

Supporting Information Table S4.docx

Supporting Information Table S1.docx

--Supporting_Information_Table_S3.docx

Supporting Information Figure S1.pdf

## Data Availability

Due to privacy and confidentiality concerns, the data underlying this study cannot be openly shared. However, research access to the data may be granted upon reasonable request to the data providers, in accordance with applicable regulations and ethical guidelines.

## References

[1] Sosialdepartementet. Perinatal care in Norway. NOU. Oslo: Universitetsforlaget; 1984.

[2] Statistisk S. Fødsler [Dataset]. [updated 2025; cited 2025 Mar 18] Oslo. Available from: https://www.ssb.no/en/befolkning/fodte-og-dode/statistikk/fodte

[3] Saunes IS, Karanikolos M, Sagan A. Norway: health system review. Health Syst Transit. 2020;22(1):1–163. 32863241

[4] Pregnancy Consultations. Available from: https://www.helsenorge.no/en/pregnancy-and-maternity-care-in-norway/antenatal-checks-and-tests/

[5] Backe B. Studies in antenatal care [PhD thesis]. SINTEF NIS Norwegian Institute for Hospital Research: University of Trondheim; 1994.

[6] From January; 2022, all pregnant women over the age of 35 will be offered NIPT and an early ultrasound [press release]. Regjeringen.no: the Norwegian Government, Nov 10 2021.

[7] Helsedirektoratet. Tilgang på og behov for jordmødre. [Online document]. Oslo: helsedirektoratet; 2021.

[8] Backe B, Jacobsen G. General practitioners’ compliance with guidelines for antenatal care. Scand J Prim Healthc. 1994;12(2):101–102. 10.3109/02813439409003683 7973187

[9] Backe B. Overutilization of antenatal care in Norway. Scand J Public Health. 2001;29(2):129–132. doi: 10.1177/14034948010290021001. 11484865

[10] Backe B. Antenatal care in Norway - many unnecessary check ups. Tidsskrift Den Norske Legeforen. 2002;122(20):1989–1992. 12555444

[11] Byhring HS, Balteskard L, Shu J. et al. Healthcare Atlas for Obstetric Services: Use of maternity services in Norway 2015–2017. Report No. 2/2019. Tromsø: Centre for Clinical Documentation and Evaluation (SKDE); 2019. Available from: https://apps.skde.no/helseatlas/files/helseatlas-fodselshjelp.pdf

[12] Folkehelseinstituttet. Medical Birth Register [Dataset]. February 4, 2024 Oslo. Available from: https://statistikkbank.fhi.no/mfr/

[13] Kinge JM, Grytten J. The impact of primary care physician density on perinatal health: evidence from a natural experiment. Health Econ. 2021;30(12):2974–2994. doi: 10.1002/hec.4426. 34498332

[14] Hansen MH, Sandbakken MH, Bjarne A, et al. Worries and information seeking during pregnancy: a cross-sectional study among 1402 expectant Norwegian women active on social media platforms. Scand J Prim Health Care. 2025;43(2):488–499. doi: 10.1080/02813432.2025.2461036. 39953944 PMC12090261

[15] Baker B, Yang I. Social media as social support in pregnancy and the postpartum. Sex Reprod Healthc. 2018;17:31–34. doi: 10.1016/j.srhc.2018.05.003. 30193717

[16] Osuide JO, Parsa AD, Mahmud I, et al. The effect of limited access to antenatal care on pregnancy experiences and outcomes among undocumented migrant women in Europe: a systematic review. Front Glob Womens Health. 2024;5:1289784. doi: 10.3389/fgwh.2024.1289784. 38379839 PMC10876992

[17] Bains S, Skråning S, Sundby J, et al. Challenges and barriers to optimal maternity care for recently migrated women - a mixed-method study in Norway. BMC Pregnancy Childbirth. 2021;21(1):686. doi: 10.1186/s12884-021-04131-7. 34620114 PMC8495671

[18] Vikum E, Johnsen R, Krokstad S. Social inequalities in patient experiences with general practice and in access to specialists: the population-based HUNT Study. BMC Health Serv Res. 2013;13(1):240. doi: 10.1186/1472-6963-13-240. 23816237 PMC3718649

[19] Austad B, Vie G, Hansen MH, et al. Association of self-rated health in pregnancy with maternal childhood experiences, socioeconomic status, parity, and choice of antenatal care providers: cross-sectional study. JMIR Form Res. 2025;9:e68811–e68811. doi: 10.2196/68811. 40460308 PMC12151455

[20] Geddes-Barton D, Baldelli S, Karthikappallil R, et al. Association between socioeconomic disadvantage and severe maternal morbidity and mortality in high-income countries: a systematic review. J Epidemiol Community Health. 2025;79(3):207–215. doi: 10.1136/jech-2024-222407. 39516003 PMC11874458

[21] Bakken IJ, Ariansen AMS, Knudsen GP, et al. The norwegian patient registry and the norwegian registry for primary health care: research potential of two nationwide health-care registries. Scand J Public Health. 2020;48(1):49–55. doi: 10.1177/1403494819859737. 31288711

[22] Behboudi-Gandevani S, Bidhendi-Yarandi R, Hossein Panahi M, et al. Prevalence of preterm birth in Scandinavian countries: a systematic review and meta-analysis. J Int Med Res. 2023;51(10):3000605231203843. doi: 10.1177/03000605231203843. 37843530 PMC10683576

[23] Ministry Of Health C Services. Time for action – personnel in a sustainable health and care service. Report No.: NOU 2023:4. Oslo: helsepersonellkommisjonen; 2023.

[24] Dowswell T, Carroli G, Duley L, et al. Alternative versus standard packages of antenatal care for low-risk pregnancy. Cochrane Database Syst Rev. 2015;2015(7):Cd000934. doi: 10.1002/14651858.CD000934.pub3. 26184394 PMC7061257

