# Supplementary figures and images for: Too much care? Increasing checkup frequencies and declining role of general practitioners in antenatal care in Norway (2010-2021)
Source: Scand J Prim Health Care. 2025 Oct 22;44(1):1–14. doi: 10.1080/02813432.2025.2575326 (PMC12918378; doi:10.1080/02813432.2025.2575326)

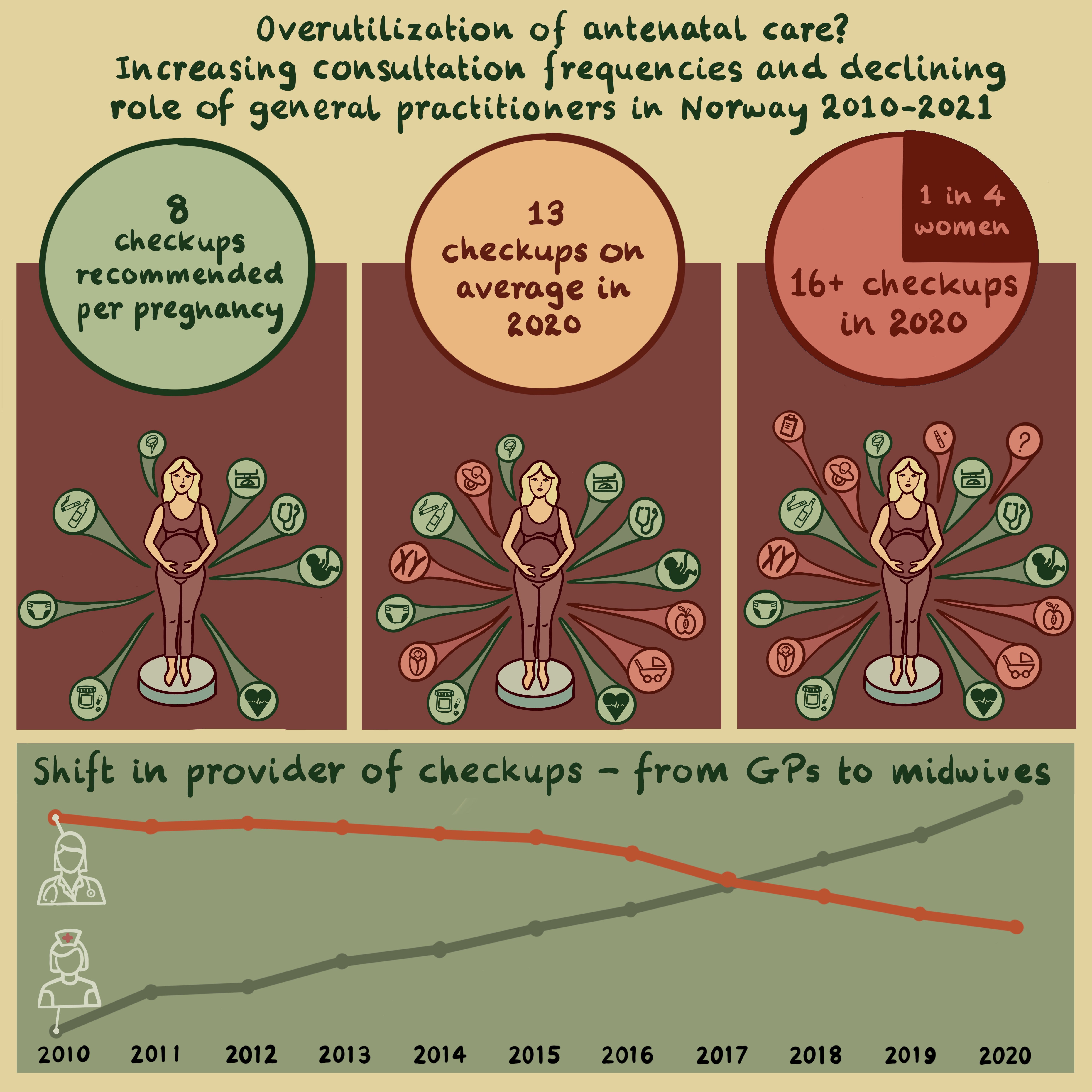

Supplement: Graphical Abstract Figure.jpg [file IPRI_A_2575326_SM4264.jpg]

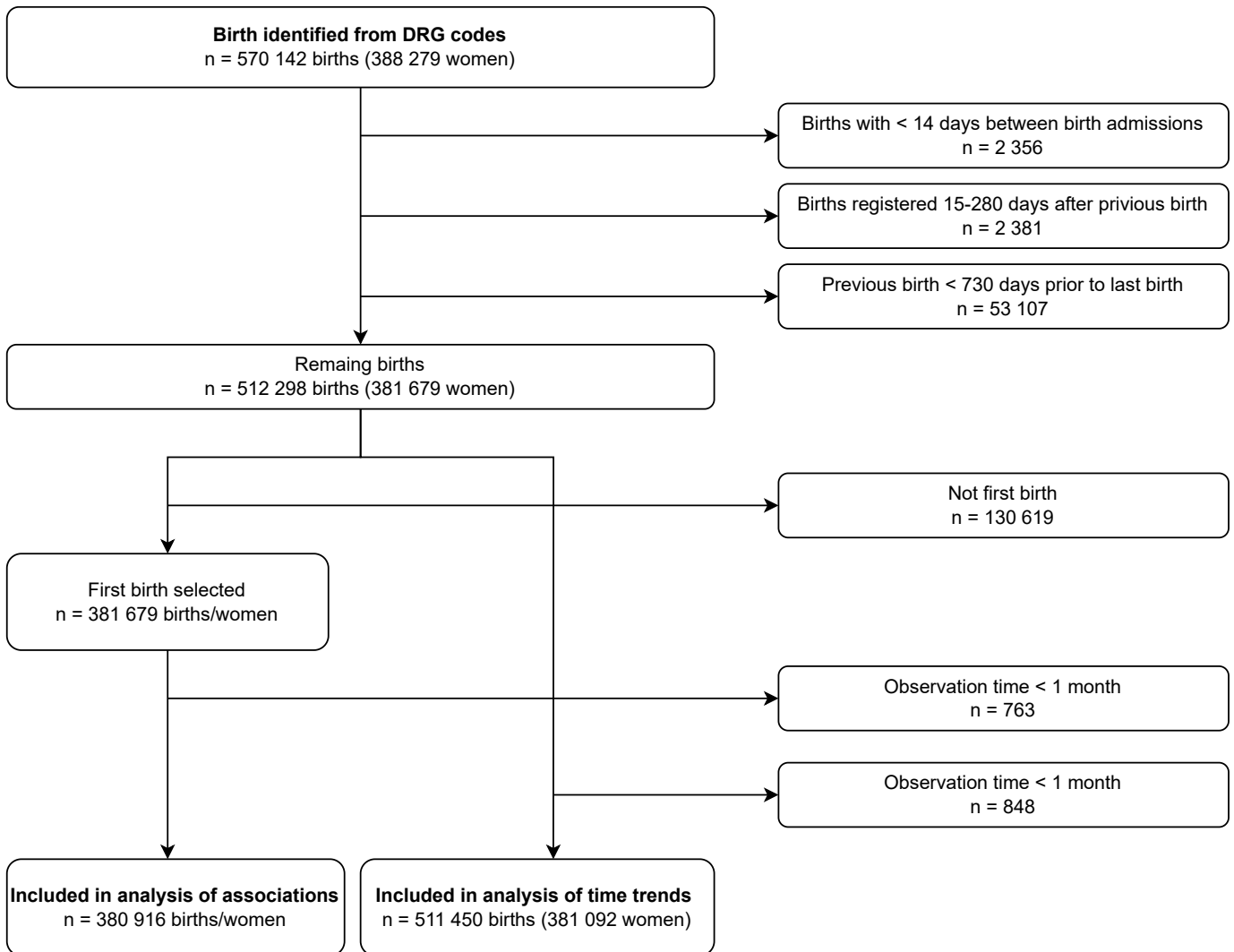

Supplement: Supporting Information Figure S1.pdf [file IPRI_A_2575326_SM4259.pdf]
