# Supplementary material for: Too much care? Increasing checkup frequencies and declining role of general practitioners in antenatal care in Norway (2010-2021)
Source: Scand J Prim Health Care. 2025 Oct 22;44(1):1–14. doi: 10.1080/02813432.2025.2575326 (PMC12918378; doi:10.1080/02813432.2025.2575326)
Supplement: Supporting Information Table S5.docx [file IPRI_A_2575326_SM4263.docx]

**Supporting Information Table S5:** Mean number of antenatal checkups with either a GP or a midwife. Presented separately for women who only saw a midwife, only a GP, or both combined as their primary care provider (irrespective of use of specialized health services), from Jan 1 2010 – Dec 31 2020.

|  |  | **Woman (n)** | **Checkups (n)** | **SD** |
| --- | --- | --- | --- | --- |
| 2010 | Only midwife | 1 478 | 5.9 | 3.0 |
|  | Only GP | 17 790 | 6.0 | 3.5 |
|  | Combination of both | 27 018 | 9.8 | 3.3 |
|  | Total | 46 286 |  |  |
| 2011 | Only midwife | 1 548 | 6.1 | 2.9 |
|  | Only GP | 13 627 | 6.1 | 3.5 |
|  | Combination of both | 28 276 | 9.8 | 3.2 |
|  | Total | 43 451 |  |  |
| 2012 | Only midwife | 1 771 | 6.2 | 3.1 |
|  | Only GP | 13 740 | 6.3 | 3.5 |
|  | Combination of both | 30 654 | 9.6 | 3.2 |
|  | Total | 46 165 |  |  |
| 2013 | Only midwife | 1 842 | 6.3 | 3.0 |
|  | Only GP | 11 875 | 6.5 | 3.5 |
|  | Combination of both | 31 706 | 9.7 | 3.2 |
|  | Total | 45 423 |  |  |
| 2014 | Only midwife | 2 061 | 6.4 | 2.9 |
|  | Only GP | 11 181 | 6.6 | 3.6 |
|  | Combination of both | 32 173 | 9.7 | 3.2 |
|  | Total | 45 415 |  |  |
| 2015 | Only midwife | 2 108 | 6.5 | 2.9 |
|  | Only GP | 10 461 | 6.7 | 3.5 |
|  | Combination of both | 33 440 | 9.8 | 3.2 |
|  | Total | 46 009 |  |  |
| 2016 | Only midwife | 2 477 | 6.7 | 2.8 |
|  | Only GP | 9 922 | 6.6 | 3.5 |
|  | Combination of both | 34 672 | 9.8 | 3.2 |
|  | Total | 47 071 |  |  |
| 2017 | Only midwife | 2 804 | 6.7 | 2.8 |
|  | Only GP | 8 576 | 6.3 | 3.3 |
|  | Combination of both | 34 678 | 9.7 | 3.1 |
|  | Total | 46 058 |  |  |
| 2018 | Only midwife | 2 808 | 6.9 | 2.8 |
|  | Only GP | 7 353 | 6.2 | 2.0 |
|  | Combination of both | 35 805 | 9.8 | 3.1 |
|  | Total | 45 966 |  |  |
| 2019 | Only midwife | 3 411 | 6.9 | 2.7 |
|  | Only GP | 6 194 | 6.1 | 3.0 |
|  | Combination of both | 35 809 | 9.8 | 3.1 |
|  | Total | 45 414 |  |  |
| 2020 | Only midwife | 3 727 | 7.2 | 2.7 |
|  | Only GP | 4 942 | 6.4 | 3.0 |
|  | Combination of both | 34 917 | 9.9 | 3.2 |
|  | Total | 43 586 |  |  |
