# Supplementary material for: Too much care? Increasing checkup frequencies and declining role of general practitioners in antenatal care in Norway (2010-2021)
Source: Scand J Prim Health Care. 2025 Oct 22;44(1):1–14. doi: 10.1080/02813432.2025.2575326 (PMC12918378; doi:10.1080/02813432.2025.2575326)
Supplement: Supporting Information Table S4.docx [file IPRI_A_2575326_SM4262.docx]

**Supporting Information Table S4:**

The utilization of GP consultations in the year prior to pregnancy, in women who were frequent users of antenatal checkups. Frequent users were defined as 16 or more checkups (upper quartile of data population). Compared to non-frequent users (0-16 antenatal checkups).

| **Utilization of GP consultations in the year prior to pregnancy in frequent users of antenatal checkups (16+ checkups per pregnancy)** | | | | | |
| --- | --- | --- | --- | --- | --- |
|  |  | **0-15 antenatal checkups** | | **16+ antenatal checkups** | |
| Mean number of GP consultations in the year prior to pregnancy | | 2.7 |  | 4.3 |  |
| Mean age |  | 30.3 | years | 30.6 | years |
| Highest level of education | Lower secondary education (13-15 yo) or lower | 17.0 | % | 22.1 | % |
|  | Upper secondary education (16-19 yo) | 24.8 | % | 27.2 | % |
|  | Bachelor's degree | 38.3 | % | 35.6 | % |
|  | Master's degree or higher | 17.6 | % | 13.5 | % |
|  | *Missing data on education* | *2.3* | *%* | *2.0* | *%* |
| Immigration status of mother | Norwegian born with two Norwegian parents | 73.0 | % | 71.7 | % |
|  | Norwegian born with one Norwegian parent | 4.5 | % | 4.5 | % |
|  | Norwegian born with immigrant parents | 1.3 | % | 1.6 | % |
|  | Immigrant | 21.2 | % | 22.2 | % |
| **Proportion of women who received the following ICPC2 diagnostic codes (grouped) from their GP in the year prior to pregnancy** | | | | | |
| A General and unspecified | | 25.9 | % | 31.7 | % |
| B Blood forming organs and Immune Mechanism | | 1.3 | % | 1.9 | % |
| D Digestive |  | 14.3 | % | 21.8 | % |
| K Cardiovascular | | 2.6 | % | 4.2 | % |
| L Musculoskeletal | | 21.2 | % | 29.2 | % |
| N Neurological |  | 7.7 | % | 11.6 | % |
| P Psychological |  | 25.1 | % | 31.9 | % |
| W Pregnancy, childbearing, family planning | | 12.5 | % | 17.1 | % |
| Other |  | 51.2 | % | 64.6 | % |
