# Supplementary material for: Too much care? Increasing checkup frequencies and declining role of general practitioners in antenatal care in Norway (2010-2021)
Source: Scand J Prim Health Care. 2025 Oct 22;44(1):1–14. doi: 10.1080/02813432.2025.2575326 (PMC12918378; doi:10.1080/02813432.2025.2575326)
Supplement: Supporting Information Table S1.docx [file IPRI_A_2575326_SM4261.docx]

**Supporting Information Table S1:**

Codes used to identify delivery date and checkups.

| **Identifying births** | |
| --- | --- |
| **DRG-codes** | |
| 370 | Caesarean section with complications |
| 371 | Caesarean section without complications |
| 371O | Caesarean section, day surgery treatment |
| 372 | Vaginal delivery with complications |
| 373 | Vaginal delivery without complications |
| 373O | Caesarean section, day treatment |
| 374 | Vaginal delivery with sterilization and/or evacuation |
| 375 | Vaginal delivery with surgery excl. sterilization and evacuation |
| 375O | Vaginal delivery with surgery excl. sterilization and evacuation, day treatment (expired) |
| **Procedural codes (NMCP and NCSP)** | |
| MAC | Amniotomy |
| MAD | Widening of the birth canal |
| MAH | Different interventions during delivery |
| MCA | Caesarean section |
| MADE00 | Ultrasound with vaginal probe during pregnancy and delivery - expired code |
| MADE10 | Abdominal ultrasound in pregnancy – expired code |
| MAF | Forceps delivery by head presentation |
| MAE | Delivery by vacuum extraction |
| MAGM05 | Induction of labor with oral prostaglandin |
| MAGM10 | Induction of labor with intravenous drug |
| MAGM11 | Stimulation of uterine contraction with intravenous drug |
| MAGM16 | Induction of labor with locally applied drug |
| MAX00 | Episiotomy |
| MAX20 | Manual intervention for head delivery |
| TMA00 | Episiotomy – expired code |
| TMA20 | Manual intervention for head delivery (expired) |
| MAG | Manual intervention for breech delivery |
| MAFE | Fetal ECG – expired code |
| TM010 | Induction of labor with unspecified intravenous drug (expired) |
| TM016 | Induction of labor with unspecified locally applied drug (expired) |
| TM612 | Induction of labor with intravenous oxytocin (expired) |
| TM613 | Stimulation of uterine contraction with intravenous drug (expired) |
| TM615 | Induction of labor with cervical prostaglandin (expired code) |

| **Antenatal checkups conducted by the primary health service** | |
| --- | --- |
| **From KUHR** | |
| W781 | Antenatal checkup (ICPC-2 code) |
| W78 | Pregnancy, confirmed (ICPC-2 code). Only included if no other ICPC-2 code was given. |
| 217a | First complete antenatal checkup and completion of pregnancy journal by GP |
| 217b | Later antenatal checkups by GP |
| 1a | First complete antenatal checkup and completion of pregnancy journal by midwife |
| 1b | Later antenatal checkups by midwife |

| **Antenatal checkups conducted by the secondary health service** | |
| --- | --- |
| **From NPR (ICD-10 codes)** | |
| Z34 | Control of normal pregnancy |
| Z35 | Control of high-risk pregnancies |
| Z36 | Prenatal diagnostics |
| O10-O16 | Edema, proteinuria and complications of hypertensive disorders during pregnancy, labor, delivery and postpartum |
| O20-O28 | Other maternal conditions, mainly related to pregnancy |
| O30-O48 | Care and treatment of the mother for fetal and amniotic conditions and possible obstetric problems |
| **From KUHR (Norwegian: “*takster*”)** | |
| 211a | Gynecological ultrasound examination of pregnant women and examination with an abdominal probe in cases of suspected pregnancy pathology and gynecological problems. |
| 211b | Ultrasound examination of pregnant women and ultrasound examination in case of suspected growth retardation (weight estimation, amniotic fluid volume assessment and respiratory movements) |
| 211e | Fetal doppler examination of high-risk pregnant women. |
| 216 | Cardiotocography (CTG) in pregnancy - "Non-stress test" |
| 217a | First complete antenatal checkup and completion of pregnancy journal by specialist |
| 217b | Later antenatal checkups by specialist |
