# Supplementary material for: Too much care? Increasing checkup frequencies and declining role of general practitioners in antenatal care in Norway (2010-2021)
Source: Scand J Prim Health Care. 2025 Oct 22;44(1):1–14. doi: 10.1080/02813432.2025.2575326 (PMC12918378; doi:10.1080/02813432.2025.2575326)
Supplement: --Supporting_Information_Table_S3.docx [file IPRI_A_2575326_SM4260.docx]

**Supporting Information Table S3:**

Sensitivity analysis: Changes in mean number of antenatal checkups from Jan 1 2010 – Des 31 2020, excluding women with high-risk pregnancies, preeclampsia, and gestational diabetes.

| **Year** | **GP consultations for any reason** | **Antenatal checkups (total)** | **Antenatal checkups by GP** | **Antenatal checkups by midwives** | **Antenatal checkups by others** |
| --- | --- | --- | --- | --- | --- |
| **2010** | 7.3 | 10.8 | 5.0 | 3.0 | 2.9 |
| **2011** | 7.3 | 11.7 | 4.9 | 3.4 | 3.5 |
| **2012** | 7.4 | 11.7 | 4.9 | 3.4 | 3.4 |
| **2013** | 7.4 | 11.9 | 4.8 | 3.7 | 3.4 |
| **2014** | 7.4 | 12.0 | 4.8 | 3.8 | 3.4 |
| **2015** | 7.4 | 12.2 | 4.8 | 4.0 | 3.4 |
| **2016** | 7.3 | 12.3 | 4.6 | 4.2 | 3.5 |
| **2017** | 7.3 | 12.4 | 4.4 | 4.5 | 3.6 |
| **2018** | 7.4 | 12.5 | 4.2 | 4.7 | 3.6 |
| **2019** | 7.5 | 12.6 | 4.0 | 5.0 | 3.6 |
| **2020** | 7.9 | 12.7 | 3.9 | 5.4 | 3.4 |
| **Mean 2010-2020** | **7.4** | **12.1** | **4.6** | **4.1** | **3.4** |
| **Change from**  **2010-2020** | **0.6** | **1.9** | **-1.1** | **2.4** | **0.5** |
